# Multiple processes in two-dimensional visual statistical learning

**DOI:** 10.1371/journal.pone.0172290

**Published:** 2017-02-17

**Authors:** Eiichi Hoshino, Ken Mogi

**Affiliations:** 1 Department of Computational Intelligence and System Science, Tokyo Institute of Technology, Nagatsuta-cho, Midori-ku, Yokohama, Japan; 2 Sony Computer Science Laboratories, Higashi-gotanda, Shinagawa-ku, Tokyo, Japan; Waseda University, JAPAN

## Abstract

Knowledge about the arrangement of visual elements is an important aspect of perception. This study investigates whether humans learn rules of two-dimensional abstract patterns (exemplars) generated from Reber's artificial grammar. The key question is whether the subjects can implicitly learn them without explicit instructions, and, if so, how they use the acquired knowledge to judge new patterns (probes) in relation to their finite experience of the exemplars. The analysis was conducted using dissimilarities among patterns, which are defined with n-gram probabilities and the Levenshtein distance. The results show that subjects are able to learn rules of two-dimensional visual patterns (exemplars) and make categorical judgment of probes based on knowledge of exemplar-based representation. Our analysis revealed that subjects' judgments of probes were related to the degree of dissimilarities between the probes and exemplars. The result suggests the coexistence of configural and element-based processing in exemplar-based representations. Exemplar-based representation was preferred to prototypical representation through tasks requiring discrimination, recognition and working memory. Relations of the studied judgment processes to the neural basis are discussed. We conclude that knowledge of a finite experience of two-dimensional visual patterns would be crystalized in different levels of relations among visual elements.

## Introduction

Humans collect information from the environment, often without conscious efforts or formal instructions [1]. In that process, humans construct knowledge of categories, whereby we can make judgment of a novel event as to whether it is a member of a group defined by previous experience [2]. The arrangement of elements is one of the features that define a category, often found in music, language and design. In the visual domain, objects in the two-dimensional (2-D) visual field consist of components that make potentially infinite combinations. Knowledge about which parts of scenes are likely to be in proximity [3, 4] and which individual scenes are classified together [5, 6] facilitate our understanding of natural scenes. However, the exact nature of the process in which humans organize initially nonsensical visual scenes into meaningful representations is not known. A key question is whether humans can construct categorical knowledge from 2-D visual arrangement alone.

The process of the learning arrangement patterns of stimuli has been studied under sequential exposures to auditory [7, 8, 9] and visual [10, 11, 12, 13, 14] stimuli. Those stimuli are abstract and initially nonsensical for subjects due to the exclusion of prior knowledge. Fiser and Aslin presented multiple scenes that contain sequences of elements over time. Their subjects exhibited sensitivity to conditional probability (i.e. *P*(A|B)) between elements (i.e. A and B) of the sequences [10, 12], where the conditional probability was calculated over the temporal dimension. These studies typically focus on the temporal frequency of multiple stimuli over time, and investigate subjects' sensitivity to this type of information. In terms of cortical processing, such analysis possibly involves the medial temporal lobe [15], where, computationally, information embedded in the mutual relations between elements is processed.

Contextual information is important in the categorical judgment of visual scenes consisting of a variety of elements projected to the retina. A scene containing some cars, lines in-between, and streetlamps probably depicts a car park: A scene consisting of a house and a few cars is likely to be a residential area. This kind of category judgment of scenes can be done almost irrespectively of feature complexity, as scene judgment occurs before the identification of features in the scenes [6]. Category representation has been modeled in two lines of theories. The prototype theory posits that categorization is accomplished by referencing to a common representation or an averaged prototype from multiple exemplars [16]. In contrast, the exemplar theory relies on the references to exemplars themselves [17, 18, 19]. In addition, there may be combined representations depending on the two approaches according to task demands [20]. In the visual domain, the nature of category representation has well been studied in the recognition of objects. Evidences suggest that both abstract category representation (not specific to a particular object) and exemplar-specific representation coexist in the left and right hemispheres, respectively [20], particularly in the fusiform cortices [21]. In contrast to object-based and feature-based representations, less is known about the representation of visual arrangement.

Effects of spatial frequency within each exemplar and collective information of multiple exemplars may arise because it is possible that exemplar-based information influences categorization, as suggested in the exemplar theory of category. Similarity of a probe to exemplars influences category judgment, automatically and mandatorily [22]. Thus, it is important to consider how learned exemplars affects judgment of a probe regarding similarity. Exemplar-based knowledge of visual arrangement would enable the subjects to voluntarily find out rules within the presented elements and attribute them to individual events [23]. A recent computational study suggests that humans may acquire such knowledge by learning parts of exemplars as well as relations between them [24].

In the actual environment, humans seldom see objects or sequences of objects in isolation. Ensembles of objects constitute a scene, with various conditional probabilities between them. Humans are sensitive to conditional probabilities of sequences in the scene [10, 12], which reflect rules that generate them. Rules are embedded in the collection of sequences and contain multiple elements with several conditional probabilities, which could be generated from a formal grammar. To the best of our knowledge, there have not been sufficient experiments which show how humans learn rules within and across scenes, and how they use the acquired knowledge in later judgments of novel scenes. Such cognitive processes may share properties with language acquisition, as both consist of elements (i.e. letters or words) with various conditional probabilities between them. The artificial grammar (AG) learning [25] is a useful paradigm to control such information and to study implicit learning. Patterns generated from AG are composed of distinct elements, which can be quantified in terms of the occurrence of frequencies known as n-gram probabilities (also known as transitional probabilities), and the Levenshtein distance [26, 27]. Studies using AG have shown that humans are able to learn rules of visual sequences along a single (spatial or temporal) dimension [14, 28]. It has been suggested that vision is better at extracting spatial order statistics than temporal order statistics [28]. Visual sequence learning was affected by element positions in sequences [28].

This study aimed to investigate whether humans can learn rules of 2-D abstract patterns (exemplars) consisting of shapes in an implicit manner without explicit instructions, and if so, how they use the acquired knowledge to judge new patterns (probes) in relation to their finite experience of exemplars.

## Methods

17 subjects (10 females and 7 males aged 18–34, with an average of 22.1 and standard deviation of 5.8) participated in this experiment. The number of subjects seems adequate to test the current hypothesis, in reference to artificial grammar studies that revealed human abilities of rule learning [9, 15, 25, 26]. All subjects had normal or corrected-to-normal vision. They were remunerated (1000 yen) for participating. They gave written informed consent after being explained about the purpose and nature of the experiments. The experimental protocol was approved by the Brain and Cognitive Sciences Ethics Committee of Sony Computer Science Laboratories.

**Fig 1:**
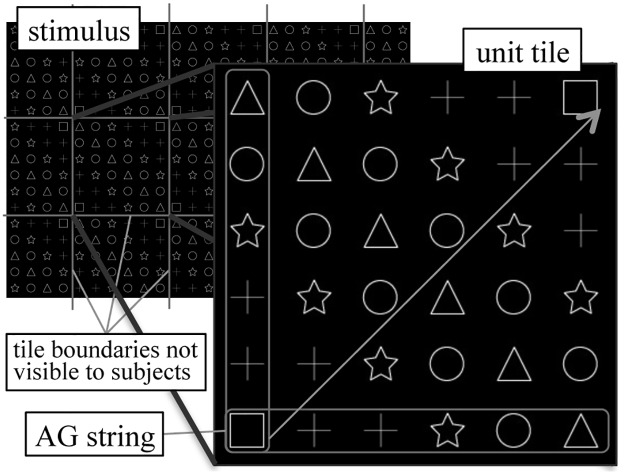
Pattern generation and an example stimulus.

An artificial grammar with five letters described in Reber's study [29] was used to generate ruled strings in this experiment. The letters T/X/V/P/S in the original study were substituted by shapes, i.e. square/plus/star/circle/triangle, respectively (Fig 1). The strings were diagonally expanded to make units of tiles so that the resulting patterns were symmetric with respect to the pi/4 and 3pi/4 lines. The units were recursively tiled to cover the computer display with a resolution of 1680 x 1050 pixels, to eliminate information regarding apparent tile edges or element positions.

The AG generated a total of 43 possible strings with lengths of up to eight, with corresponding visual patterns. For each subject, 25 patterns (exemplars) were randomly chosen to represent all paths through the AG for the study phase, while the remaining 18 (probes) were reserved for the test phase. As a control to the AG, 43 strings were randomly generated for each subject using the same set of shapes, matching the AG strings in length. They were converted to visual patterns in the same procedure as the AG generated patterns.

The patterns were presented on a computer display, which was placed at a distance of approximately 60 cm from the subjects.

The experiment consisted of a study phase followed by a test phase.

For the study phase, 25 learning patterns (exemplars) were randomly divided into 5 sets, each containing 5 patterns for each subject. The number of patterns was set to be 5, based on a pilot study which indicated that most subjects completed the study phase in the first two consecutive sessions with four patterns, but did not with five patterns, possibly due to the limited capacity of visual short-term memory storage depending on stimulus complexity [30].

Much of the procedure in the study phase was adapted from Reber’s work on AG [25], which required the subjects to reproduce nonsensical words, with two consecutive correct reproductions required to proceed to the next set. In the current experiment, after the presentation of each pattern in a set, the subjects were instructed to answer the order of presentations, instead of drawing up the patterns. This procedure was designed to control familiarities of patterns at the same level [25], as well as to keep the subjects' attention to the patterns [28]. The control of familiarities was particularly important for exemplar-based analysis, assuming familiarities of exemplars were the same.

In the study phase, the subjects viewed a blank for 100 ms, a number indicating the order of presentation for 1 s, and 1 out of 5 patterns (exemplars) for 5 s. This procedure was repeated 5 times without breaks to complete the 5 patterns of a set. The subjects then viewed one of the 5 patterns. They were instructed to answer the order of the presentation by pressing a number key from 1 to 5. After answering, the subjects viewed the next pattern following a 100 ms blank, until the completion of 5 patterns. These procedures constituted a single trial. No feedback of correct/wrong was given. Trials with the same stimulus set with shuffled orders for presentations and questions were repeated until the criterion of two consecutive correct answers for all 5 orders was reached. When one set was completed, a new set of 5 patterns was learned until all 5 sets were finished.

For the test phase, a set consisting of 79 patterns (probes) was prepared, which included 18 AG generated patterns (not used in the study phase) twice each, and 43 control patterns. The 79 patterns with shuffled orders were presented one by one until the subjects responded. The subject’s task was to answer whether the rule of a pattern presented was the “same” or “different” compared with the rule for previously learned 25 patterns in the study phase, in a two-alternative forced-choice procedure. The subjects were specifically instructed as follows: “The 25 patterns you have seen were based on a rule. From now on, patterns will appear on the screen one by one. Please answer by pressing a key whether the pattern is based on the same rule or a different one.” No explicit remark about the construction of the rules was given, in order not to interfere with the subjects’ own conceptions about the nature of patterns. The subjects were instructed to place their index fingers on the “f” and “j” key as home positions. Half of the subjects were instructed to press the “f” key if they felt a pattern presented was “same” and the “j” key if “different”, while the other half was instructed vice versa in a counterbalance.

The subjects were initially informed only about the study phase and not about the test phase, to avoid explicit categorization or rule searching when they tackled the study phase. After finishing the study phase, they were given instructions about the test phase. After completing computer-based tasks, they answered a written questionnaire about the experiment.

## Analysis

The analysis was based on objective measures rather than predefined rules. In the categorical judgment of visual arrangements, similarities among exemplars (patterns in the study phase) and probes (patterns in the test phase) have been a particularly interesting subject for research [22]. To analyze similarities among patterns, we introduced dissimilarity measures reflecting relations between elements, namely the Levenshtein distance (hereafter LD) and n-gram probabilities (also known as transitional probabilities). LD is defined as the minimum number of operations (deletions, insertions and substitutions) required to convert one sequence into the other [27]. We applied LD to analyze the relations of elements in the seed strings of the patterns, which were in the bottommost row and the leftmost column of tiles. In addition, n-gram probabilities were used to measure the 2-D relations of elements in tiles. An n-gram probability represents a probability of the occurrence of an item conditioned on its n-1 contiguous items (i.e. *P(x*_*i*_ *| x*_*i-(n-1)*_, *…*, *x*_*i-1*_*)*) [26]. We defined an n-gram dissimilarity of pattern A compared with pattern B as follows. First, we picked up any n-grams from the unit tile of pattern A. Next, we calculated n-gram probabilities for each of these n-grams in the unit tile of pattern B. Finally, we obtained an n-gram dissimilarity as 1 minus the mean of the n-gram probabilities (i.e. 1—∑ *P*_A_(*x*_*i*_,*x*_*i-1*_,*…*,*x*_*i-(n-1)*_) × *P*_B_(*x*_*i*_ *| x*_*i-(n-1)*_, *…*, *x*_*i-1*_) (*x* ∈A, B), refer to S1 Fig for an example). We calculated dissimilarities for 1-, 2- and 3-gram, taking the probes as A and the exemplars as B in the aforementioned equation. Dissimilarities for n-grams with n>3 were not calculated because most of n-grams in a pattern of more than 3 sequences are not found in another pattern when n>3. A 1-gram probability had no conditional probability (i.e. *P(x*_*i*_*)*) and was equal to the mean frequency of five elements. In the 2- and 3- grams, every contiguous sequence was taken from a unit tile to calculate possible combinations of n-grams. Note that the resulting n-grams are the same regardless of whether the contiguous sequence was taken horizontally or vertically, because of the symmetry of patterns along the pi/4 and 3pi/4 lines. If an element of interest is near an edge of a unit tile and its conditional elements are outside of the tile, the conditional probability was defined with the outside elements as the neighbor tiles. The 3-gram was derived from averaging two ways of calculations depending on how conditional elements were assigned, i.e., conditional-conditional-target and conditional-target-conditional.

The dissimilarity measures reflect context-dependency or levels of relations among elements [31]. 1-gram is a context-independent measure within patterns, and thus is an element-based processing of the patterns, defined as the frequency of each element regardless of its spatial configuration. N-grams with larger N implicates more context-dependency. Thus, 2- and 3-grams are based on configural processing, reflecting spatial configural relations among multiple elements within patterns. LD is also considered to be based on configural processing, because editing an element requires positional information defined relative to non-target elements. For example, consider a case in which the string PPXS is converted to the string TXS. To calculate LD, one would first need to compare these two strings, and arrive at a single sequence, allowing for the possibilities of insertions or deletions. In this case, the last two letters XS are the same. Next, one needs to know where different letters are located relative to XS. In this case, they are on the left of X. After replacing P with T or deleting P, one would still need to handle one more P located the leftmost, to be deleted or replaced with T. These manipulations would necessarily involve relations among multiple elements.

In addition, to investigate how the knowledge of multiple familiarized exemplars is related to subjects' “same” and “different” judgments in the test phase, we applied three alternative analyses of dissimilarity measures. 1) As exemplar-based analysis, dissimilarities for each probe compared with each of 25 exemplars were calculated. Those dissimilarities were sorted in an ascending order for each probe so that 25 exemplars were ranked in the order of similarity. For instance, a rank 1 exemplar of LD was the most similar to, or the least distant from a particular probe in the measure of LD. For each of the 61 probes in the test phase, the 25 exemplars were given the ranking of 1 to 25. When two exemplars had the identical dissimilarity measure, they were assigned to consecutive ranks for the convenience of further analysis. The probes were put into two ensembles, depending on whether the subjects classified them as “same” or “different”. As the 18 AG generated probes were presented twice each, they were sorted twice into the respective ensembles, with possibly inconsistent responses from the subject. Finally, dissimilarity measures between the probes and ranked exemplars (a particular exemplars given a specific rank depending on the probe) were averaged over ranks and subjects for the “same” and “different” ensembles. 2) The mean dissimilarities of all 25 exemplars were calculated, which were equal to the average of dissimilarities for all ranks. The calculations were irrespective of the interaction between ranks and judgments, conveying alternative information about knowledge regarding the average distance strategy [32]. 3) Dissimilarities for each probe compared with the most prototypical exemplar were calculated. The most prototypical exemplar was defined as the exemplar with the least average dissimilarity to other 24 exemplars. Here, dissimilarity measures were calculated using the equation 1—∑ *P*_A_(*x*_*i*_,*x*_*i-1*_,*…*,*x*_*i-(n-1)*_) × *P*_B_(*x*_*i*_ *| x*_*i-(n-1)*_, *…*, *x*_*i-1*_) (*x* ∈A, B), with A taken as the exemplar in question and B taken as 24 other exemplars. The prototypical exemplar was considered to share the most attributes with the most typical member of exemplars [16].

**Fig 2:**
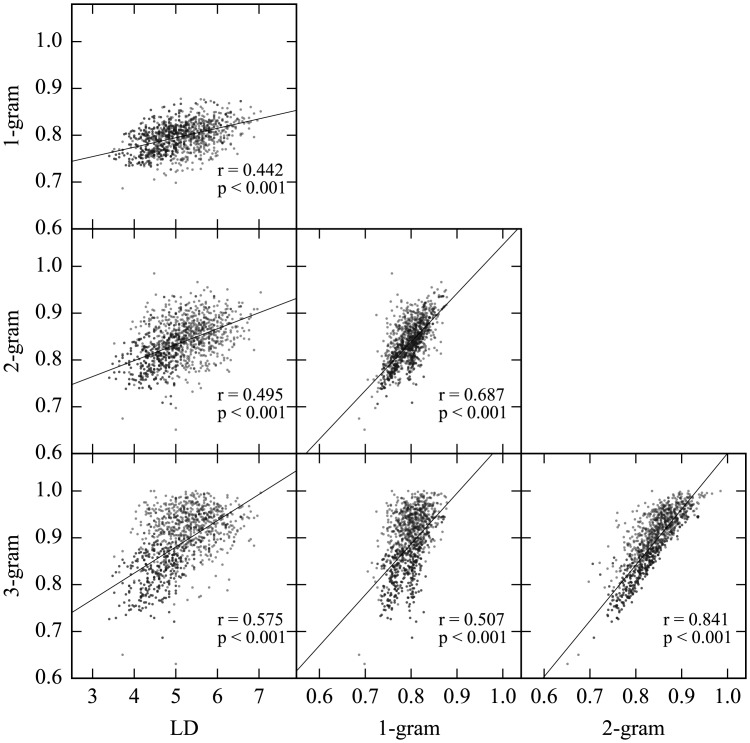
Pearson's correlation coefficients among four measures of the mean dissimilarities. The four dissimilarity measures are positively correlated.

Although these dissimilarity measures grasp different aspects of patterns, they would also share some common features. The mean dissimilarities were all significantly correlated with each other (Fig 2). In addition, we separately performed statistical tests on these dissimilarities to capture various aspects of the stimuli in each measure of relation among elements. Because the weights of these dissimilarities on “same” and “different” judgment were not known, we conducted the analysis focused on each dissimilarity rather than one between measures of dissimilarity as multiple regressions.

## Results

One subject was excluded from further participation in the experiment for not finishing the study phase within one and half an hour. 16 subjects completed the tasks. None of the subjects reported remembering any single patterns or unit tiles precisely.

**Fig 3:**
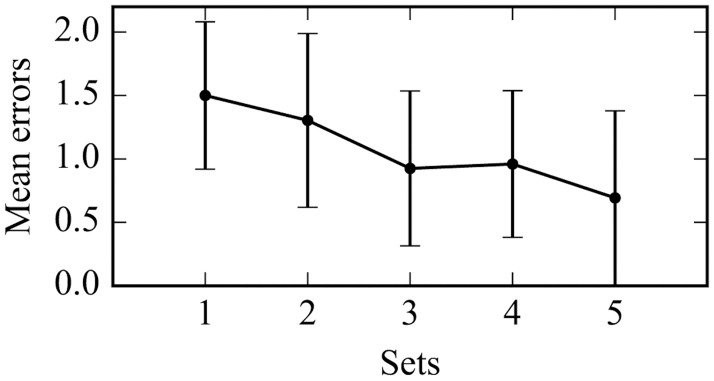
Mean number of errors out of 5 sets tested in the study phase. The error rates represent the overall average until the criterion of two consecutive correct answers for all 5 orders was reached. The error bars indicate standard deviations.

For the study phase, Page’s L test [33] revealed that the mean number of errors in the questions of the orders in a set of 5 patterns had a statistically significant descending trend in proportion to the number of sets (*p* = .0003) (Fig 3). Post hoc tests using the Bonferroni correction revealed that number of sets elicited a slight reduction in the mean errors from Set 1 (1.50 ± .58) to Set 3 (.93 ± .61, *p* = .041) and 5 (.69 ± .69, *p* = .052).

For the test phase, we calculated the d-prime with a "hit" defined as a “same” judgment on AG generated patterns and a “false alarm” as a “same” judgment on control patterns. As the 18 AG patterns were presented twice each, they were counted twice into "hit" and "false alarm" in a respective manner, with possibly inconsistent responses from the subject. A one-sample t-test across subjects revealed that the mean d-prime between AG generated and control judgment was significantly above zero (Mean = .45, *T*(15) = 4.84, *p* = .0002, Cohen’s *d* = 1.21). Thus the subjects were able to discriminate between the AG generated and control patterns in the test phase. The results indicate that the subjects successfully learned aspects of the rules under the 2-D patterns implicitly, while the explicit instruction was to learn the order of presentations.

**Fig 4:**
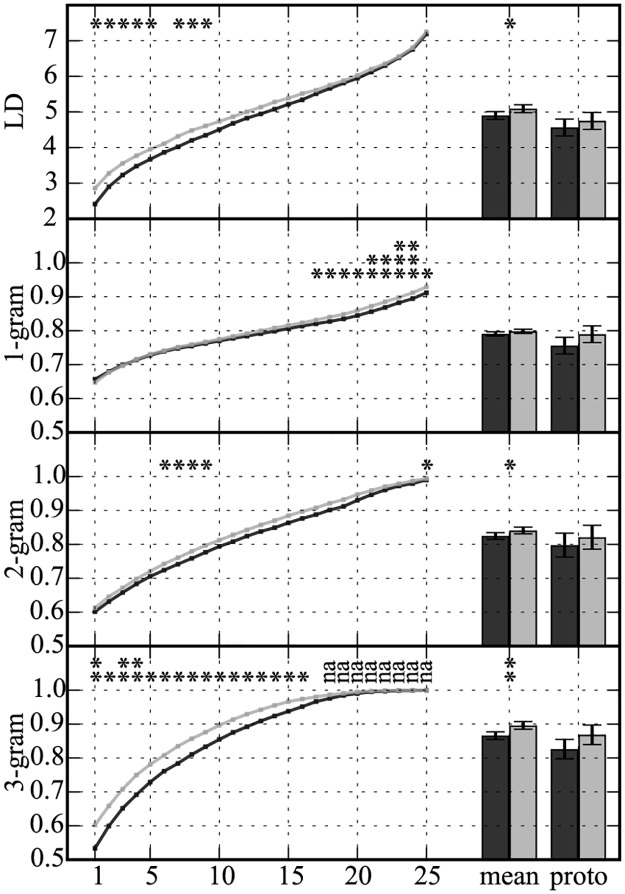
Difference between “same” and “different” judgments in four measures of dissimilarity: The x-axis indicates ranks, the mean of 25 ranks and the prototypical. Rank 1 represents the exemplar most similar to the probe, while rank 25 represents the least similar. The y-axis indicates the mean dissimilarities between exemplar and probe patterns among the subjects. The black and gray line/bars are for the “same” and “different” judgments, respectively. The asterisks indicate statistically significant differences in dissimilarity measures between “same” and “different” judgments in multiple paired t-tests with Bonferroni correction (where * p < .05, ** p < .01, *** p < .001, with the asterisks vertically represented in the graph). Error bars indicate the standard deviations. No statistical tests were performed on ranks labeled "na" due to the ceiling effect, where the dissimilarity measure took the saturated value of 1 for at least one subject. See S1 Table for statistical values.

Next we looked at the nature of the acquired knowledge in regard to the similarity between the exemplar and probe patterns (Fig 4). For each of four dissimilarity measures (i.e. LD, 1-, 2- and 3-gram), we conducted two-way repeated measures ANOVA on the degree of dissimilarities between the exemplar and probe patterns to examine the effect of similarity rank (see Analysis) and “same”/“different” judgment. There were statistically significant interactions between the effects of similarity rank and “same”/“different” judgment for all four measures of dissimilarity (i.e. LD, 1-, 2- and 3-gram) (*F*(24, 360) = 9.457, 8.943, 1.627, 15.26; *p* < .0001, *p* < .0001, *p* = .03, *p* < .0001, respectively). Further, we performed multiple paired t-tests between “same” and “different” judgments for each rank, in each of the four measures of dissimilarity. We also performed paired t-tests between “same” and “different” judgments for the mean of all examplers and the prototypical exemplar (see Analysis) altogether. We applied Bonferroni correction of n = 27 for obtained p-values, as a conservative approach. The results showed that in LD, there were significant differences in the degree of dissimilarities between “same” and “different” judgments for ranks 1 to 5, 7 to 9, and the mean (“same” < “different” with *p* < .05 and Cohen’s *d* > 0.8, for more details, see S1 Table). In 1-gram, there were significant differences in the degree of dissimilarities between “same” and “different” judgments for ranks 17 to 25 (“same” < “different” with p < .05 and Cohen’s d > 0.8). The results for 2-grams showed that the degree of dissimilarities had significant differences between “same” and “different” judgments for ranks 6 to 9, 25, and the mean (“same” < “different” with p < .05, Cohen’s d > 0.8). In 3-gram, there were significant differences in the degree of dissimilarities between “same” and “different” judgments for ranks 1 to 16, and the mean (“same” < “different” with p < .05, Cohen’s d > 0.8). We also performed the Kolmogorov-Smirnov tests for normality for each distribution of the degree of dissimilarity and found no violation of normality (S1 Table). Taken together, across the measures of dissimilarity, there were variations regarding ranges of ranks in which the subjects’ “same” and “different” judgments were related to the degree of dissimilarities between the exemplar and probe patterns.

**Fig 5:**
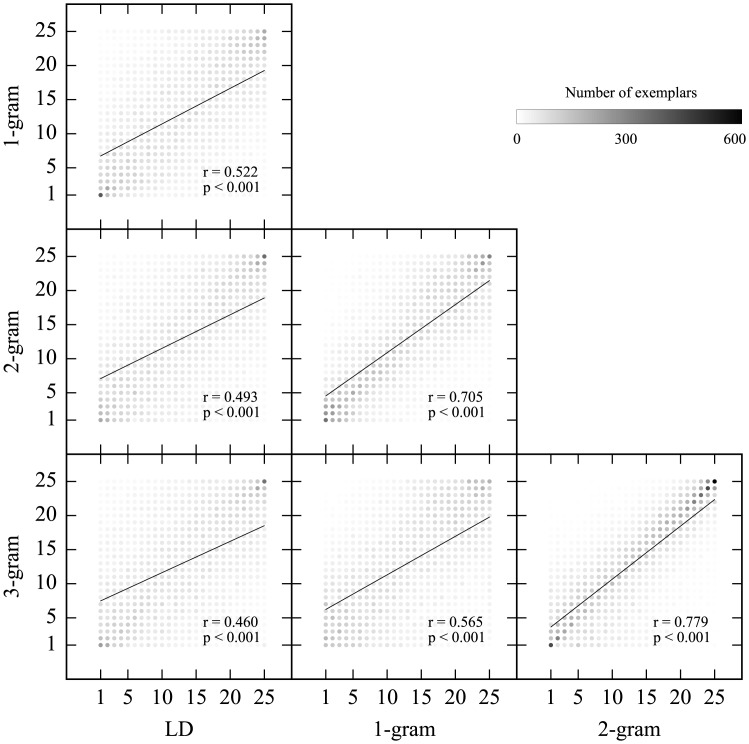
Spearman’s rank correlation coefficients among ranks in the four measures of dissimilarities. Gray scales indicate numbers of exemplars for each dot. The x and y axes are ranks of each dissimilarity.

It is possible that these variations reflect the same phenomenon viewed from different perspectives due to the nature of the current stimuli, such that the rank numbers of 1-gram was inversely correlated with that of 3-gram. To verify this point, we further calculated correlation coefficients of rank numbers, which each exemplar scored, among the measures of dissimilarity. All pairs of the measures of dissimilarity showed significant positive correlations of rank numbers (Fig 5), indicating that the variations across the measures of dissimilarity arose from the nature of subjects' judgments beyond that of simple inverse correlations.

## Discussion

We used a visual AG paradigm [25] to investigate whether human subjects can extract statistical regularity of 2-D patterns, when the explicit instruction (i.e. to memorize the presentation order) was not about the learning of regularity. AG learning approach to implicit rule extraction has been validated in previous studies, which showed that statistical learning occurs in familiarization [7, 9, 26], including one-dimensional visual patterns [14, 25, 28], visual temporal orders [11, 13] and temporal frequencies of visual spatial configurations [10, 12]. Neural correlates of visual statistical learning indicate that statistical learning occurs implicitly with little exposure to stimuli, independent of subsequent explicit familiarity [15]. These studies would suggest that humans are able to extract statistical regularity of 2-D patterns. Consistent with previous studies [7–15, 25, 26, 28], this study showed rule extraction over 2-D patterns in human subjects as demonstrated by the result of significant learning effects in the study phase (Fig 3) and positive d-prime value. Regarding the AG learning of the visual-spatial format, the current experiment is an extension of a previous study, in which Conway and Christiansen showed that humans could learn rules from horizontally displayed visual sequences that were generated by AG [28]. Their study used horizontally displayed one-dimensional sequences and found that the rule learning was affected by elements at the left end of the sequences. This effect was excluded in the current experiment by using tiled patterns in which only the spatial relations between elements were relevant. Thus, it is suggested that humans are able to learn rules in spatial relations between elements without explicit reference to specific element-position relations, at least in the case of 2-D arrangement.

The small value of d-prime between AG generated and control patterns indicates that there is possibly no fine-grained categorization according to the predefined rules. The predefined rules are not derivable precisely from the limited number of exemplars, as arguments concerning poverty of stimulus often suggest in formal language theory [34, 35]. In the current study, the subject successfully made categorical judgment (“same” or “different”) of probes: Categorization might proceed by the subjects' individual definitions based on their own experience of exemplars [36]. In the study phase, learning was unsupervised, where the rule extraction occurred in implicit learning in a task in which discrimination, recognition and working memory are required for the presentation order task without explicit instructions about rule extraction. Previous studies showed that unsupervised category learning occurs automatically or spontaneously during exposure to visual objects [37, 38]. The current experiment demonstrated that unsupervised category learning also occurs during discrimination of 2-D visual arrangement. Although unsupervised category learning typically involves ill-posed problems and demands conjecture or instinct to learn meaningful categorical knowledge [39], it has been suggested that instinctive learning or reasoning has validity [36]. The nature of subjects' judgments observed in the current study is in line with its validity, shedding light on the nature of human perception of the visual arrangement.

Importantly, the subjects' “same” and “different” judgments were related to the degree of dissimilarities between the exemplar and probe patterns (Fig 4), indicating that subjects were sensitive to the similarity between probe and exemplar patterns. It is possible that there are several aspects of cognition reflected by alternative dissimilarity measures (LD, 1-gram, 2-gram, and 3-gram). The subjects were able to base the judgment (“same” or “different”) on the probes based on exemplars most similar to the probes as represented by LD and 3-gram, two measures with high context-dependency. This tendency was also moderately exhibited in characteristics represented by 2-gram. These characteristics possibly reflect the nature of configural processing in the subjects. On the other hand, in terms of characteristics represented by 1-gram, the subjects were shown to be sensitive to information derived from exemplars least similar to the probes. These results, taken together with the result of positive correlations of rank numbers between the measures (Fig 5) would suggest that element-based and configural processing coexist in the processing of visual arrangement. This result is consistent with a lesion study suggesting the notion that local and global processing are separable in visual perception [31], and a study indicating human sensitivity to single elements and sequence of elements extracted from scenes [10]. The subjects in the current study seemingly utilized these processing to judge new patterns implicitly, while they were not necessarily conscious of what they remembered according to the post-experiment report, in line with AG learning studies [25, 29]. It is suggested that the processing of visual arrangement can be conducted implicitly, with parallel processes regarding how many elements are taken into account at once.

Reed's categorization strategies [32] explain the characteristics of element-based and configural processing. He documented four important strategies of subjective categorization; prototype, proximity algorithm, cue validity and average distance. The average distance strategy entails judgment based on the mean distances between a probe and all exemplars. In his study, the average distance strategy, as well as the prototype strategy, explained subjects' behavior in categorizing multidimensional faces. The mean dissimilarity of 25 ranks (Fig 4) in the current analysis is equivalent to the average distance strategy. The proximity algorithm could work within the framework of the exemplar theory. It predicts that judgment is based on the most similar exemplar to a probe, which is equivalent to the k-nearest neighbor (k-NN) method. The K-NN method is one of useful computational models of pattern recognition, where k in k-NN represents the number of exemplars taken into account for a given classification. In the current analysis, the subjects’ sensitivity to characteristics derived from exemplars with high ranks (most similar to the probes) and the mean dissimilarity of 25 ranks represented in LD and 3-gram, helps to explain the characteristics of highly context-dependent or informative configural processing. The subjects’ judgments may be primarily based on the proximity or the k-NN algorithm strategy in configural processing. On the other hand, the subjects’ sensitivity to dissimilarities in exemplars with low ranks (least similar to the probes) in element-based processing is similar to distal algorithm. Therefore it is possible that judgment is based on the elimination of highly dissimilar exemplars regarding element-based processing. These possibilities can be proposed on the premise that each dissimilarity analysis is separately discussed. All the measures of dissimilarity are positively correlated (Fig 2). Exemplars in high ranks in LD and 3-gram would be different from those in low ranks in 1-gram (Fig 5). Further studies will be necessary to elucidate issues concerning which strategy most contributes to judgment.

Effects of dissimilarity on judgment of new patterns were larger in 3-gram than 2-gram in which exemplars in a fewer number of ranks (rank 6 to 9) showed significant differences (Fig 4). This discrepancy between 2- and 3-gram was possibly due to the fact that characteristics of rules was more effectively represented in 3-gram than 2-gram, while the memory of embedded sequences in larger spatial configurations was inhibited [12]. There were few common n-grams for n> = 4 between patterns in the current experiment. Thus, 3-gram was the largest informative sequence in this context, whereas 2-gram was less informative. 2-gram not embedded in 3-gram might have contributed to the significant differences observed in the result of 2-gram. Fiser and Aslin asked their subjects to judge familiarity of a single sequence of elements embedded in scenes [10, 12]. The subjects were able to remember element sequences better with perfect conditional probability *p* = 1.0, compared to non-perfect conditional probability *p* = 0.5 or 0.66, when those two types of sequences were presented equal times. The current study additionally indicates that humans are sensitive to various conditional probabilities between elements of spatial sequence.

In contrast to many studies in statistical learning which have focused on temporal frequencies, the current study investigated spatial frequency of element sequences within patterns. As a result, we were able to analyze categorical judgment based on relations between probes and exemplars, keeping knowledge of individual exemplars [23]. The analysis was extended to comparison and accumulation of exemplars, reflected in ranking and the mean dissimilarity of 25 ranks, respectively. The subjects repeatedly learned each exemplar through within-category discrimination, until they reached a certain learning criteria. Accordingly, we could assume that the subjects were familiarized to the exemplars equally.

The results suggest that no prototypical representation was constructed, where there were no relation between the subjects’ “same” and “different” judgments and the degree of dissimilarities between the prototypical exemplar and probe patterns (Fig 5). It is possible that the prototypical exemplar, which is the most similar to other exemplars on the average, does not represent the actual prototype [16]. The prototypical approach assumes that a generalized knowledge is formed in category learning, whereas the exemplar approach requires memory of individual exemplars. Both approaches have advantages depending on the nature of the task [19, 40]. Briscoe and Feldman showed that humans perform a middle point of both extreme approaches in a supervised category learning with multiple feature dimensions [41]. They claimed that prototype and exemplar models are in a trade-off relationship, the former starting from a simple conceptual model while the latter had high variance to fit any predefined rules. The current result is in favor of exemplar-based representation as shown in the subjects’ sensitivity for several ranks across measures of dissimilarity (Fig 4). These findings are consistent with natural language categories [17] and evidence from neural data [18].

The mean dissimilarity of 25 ranks, was a product of the collective information of relations between exemplars and probes [32]. This computation necessarily involves exemplar-based representation because it is necessary to calculate 25 dissimilarities between probe and exemplar patterns in each rank before calculating the mean of dissimilarity. Thus the significant differences observed in the mean dissimilarity of 25 ranks indicate the existence of exemplar-based representation. It is possible that the mean dissimilarity measures reflect a more abstract category representation of multiple exemplars in contrast to exemplar-specific representation [21]. A study investigating neural correlates with a visual identification task demonstrated that abstract category is represented in the left occipital cortex and IT, while specific exemplars are represented in the right occipital cortex and IT [20]. More specifically, the core areas of abstract category representation and exemplar representation may be left and right fusiform gyri, respectively [21]. Garoff et al. [21] showed that specific minus non-specific recognition and non-specific recognition minus forgetting are associated with activities in the right and left fusiform gyri during encoding, respectively. In their study, subjects viewed and judged presented visual objects, choosing from three alternatives, "same", "similar" or "new", with respect to knowledge in a prior study phase, which was conducted in a very similar manner to the current experiment. They designated a "same" response to a "same" object as specific recognition, a "same" response to a "similar" object or a "similar" response to a "same" object as non-specific recognition. The present results are consistent with the view that exemplars contributing to strong exemplar-based representation lead to specific recognition accompanied by the right fusiform activation while exemplars contributing to abstract category representation such as average distance lead to non-specific recognition accompanied by the left fusiform activation. In addition, the characteristics of exemplars would be already determined by the fusiform cortices in the study phase [21].

Humans understand global as well as local relations [31, 42]. It is, however, not known whether spatial statistical arrangement is processed in a similar manner to temporal one, engaging brain areas associated with episodic memory [15]. The global and local processing of visual input shows some similarity to temporal statistics regarding how animals tend to process [43, 44], and thus some shared mechanisms would be involved. One region possibly involved in spatial arrangement processing is the fusiform cortex [44]. Extensive familiarization facilitates categorical selectivity in the fusiform. Not only faces but also objects of visual expertise activate the lateral side of the fusiform, also known as the fusiform face area [45]. Extensive training of tool-like novel objects elicits focal activation of the medial fusiform gyrus, a region known to be tool-selective [46]. These familiarization effects indicate that the fusiform may aggregate information of objects and categorize according to their statistics of features. On the other hand, the perirhinal cortex (PRC) plays a prominent role in discrimination between semantically similar objects [47] and between objects in the context with high degree of feature ambiguity [48]. Thus, it is possible that the fusiform cortex is involved during familiarization, such as in the study phase in this study, whereas PRC is involved when decision is required, such as in the test phase.

Finally, the results of the current study suggest the existence of element-based and configural processing in visual arrangement in humans, which is consistent with a computational study [24]. The co-existence of them in exemplar-based representation suggests that visual representation would be distributed along two axes, spatial relations within exemplars and multiple individual exemplars. The spatial axis is responsible for levels of processing, from the element-based to the configuration of multiple elements within each exemplar. This axis reflects the online analysis of spatial and perceptual information. The axis of multiple individual exemplars is for categorical knowledge, and is subserved by several factors from a single exemplar (exemplar-specific representation) to conjoint representation of multiple exemplars (abstract category representation). Categorical knowledge of exemplars involves the memory system and serves as the basis for judgment of forthcoming events. Although it is possible that there are other axes or measures that capture better aspects of visual representation, this objective analysis sheds light on human judgment of visual arrangement regarding exemplar-based representation. Specifically, the current analysis provides the evidence of both axes within a single experiment.

## Conclusion

This study shows that humans are able to learn rules of 2-D arrangement in a statistical manner. The rules contain categorical knowledge that is dominated by exemplar-based representation, and is used in later judgment of new patterns.

## Supporting information

S1 Fig An example of dissimilarity derivation between two patterns. (PDF)

S1 Table T-value and Cohen’s *d*, and p values of the Kolmogorov–Smirnov test. (PDF)
