# Supplementary material for: Multiple processes in two-dimensional visual statistical learning
Source: PLoS One. 2017 Feb 17;12(2):e0172290. doi: 10.1371/journal.pone.0172290 (PMC5315298; doi:10.1371/journal.pone.0172290)
Supplement: S1 Fig — (PDF) [file pone.0172290.s001.pdf]

An example of dissimilarity derivation

Unit tile of pattern A | neighboring pattern

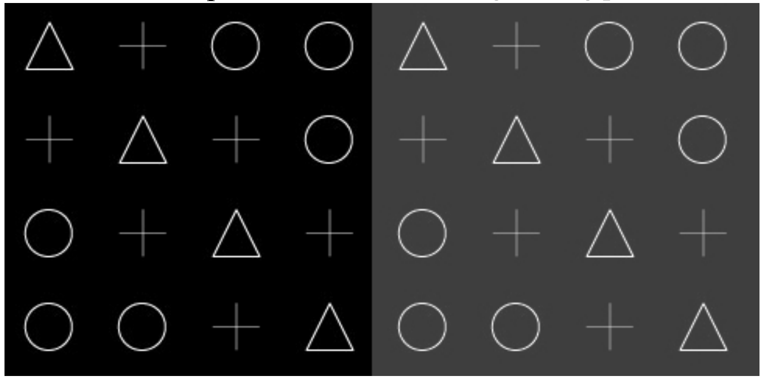

Unit tile of pattern B | neighboring pattern

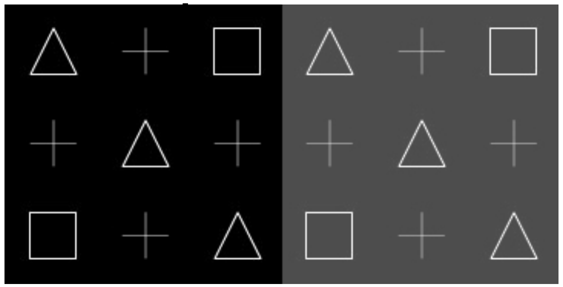

Consider calculating 2-gram dissimilarity of A compared to B. For the convenience of explanation, here we take up a case of △ +. There are 3 sequences of △ + in pattern A unit. Likewise, every 2-gram in A was counted (the third column at 2-gram in the right table). We took n-grams matching the size of the unit, with an extension to the neighboring tile. The total of 2-grams in A were 16. In pattern B, there are 2 sequences of △ + and 3 sequences that had △ at the conditional position. 2-gram probability of △ + was 2/3 in B (the conditional position being 1=n-1 (where n=2). In this case there is △ at the conditional position). Likewise, every 2-gram probability in B was calculated (the forth column). By summing of every multiplication of proportion in A and 2-gram probability in B, we obtained the mean of 2-gram probabilities regarding how frequently 2-gram appeared in A (the sixth column). Dissimilarity is obtained as 1 – the sum.

	element(s)	(a) proportion of element(s) in A	(b) (conditional) probability in B	(a) x (b)	sum of (a) x (b)	dissimilarity (1-left column)
LD						2
1-gram	□	0/16	2/9	0	0.25	0.75
	+	6/16	4/9	0.167		
	△	4/16	3/9	0.083		
	○	6/16	0/9	0		
2-gram	○ ○	2/16	0/0	0	0.21875	0.78125
	○ +	3/16	0/0	0		
	○ △	1/16	0/0	0		
	△ ○	1/16	0/3	0		
	△ □	0/16	1/3	0		
	△ +	3/16	2/3	0.125		
	+ △	3/16	2/4	0.09375		
	+ ○	3/16	0/4	0		
	+ +	0/16	1/4	0		
	+ □	0/16	1/4	0		
	□ △	0/16	1/2	0		
	□ +	0/16	1/2	0		
3-gram	○ ○ +	1/16	0/2	0	0.0	1.0
	○ ○ △	1/16	0/2	0		
	○ + △	3/16	0/1	0		
	○ △ +	1/16	0/1	0		
	+ ○ +	2/16	0/2	0		
	+ ○ ○	1/16	0/2	0		
	△ + ○	3/16	0/1	0		
	△ + +	0/16	1/2	0		
	△ + □	0/16	1/2	0		
	△ ○ ○	1/16	0/1	0		
	△ □ +	0/16	1/1	0		
	+ △ □	0/16	1/2	0		
	+ △ ○	1/16	0/2	0		
	+ △ +	2/16	1/2	0		
	+ + △	0/16	1/1	0		
	+ □ △	0/16	1/1	0		
	□ + △	0/16	1/1	0		
	□ △ +	0/16	1/1	0		
