# Supplementary material for: Multiple processes in two-dimensional visual statistical learning
Source: PLoS One. 2017 Feb 17;12(2):e0172290. doi: 10.1371/journal.pone.0172290 (PMC5315298; doi:10.1371/journal.pone.0172290)
Supplement: S1 Table — (PDF) [file pone.0172290.s002.pdf]

Table S1.

T-value and Cohen's *d*.

Rank		1	2	3	4	5	6	7	8	9	10	11	12	13	14	15	16	17	18	19	20	21	22	23	24	25	mean	proto
LD	T(15)	4.968	4.477	4.421	4.567	4.168	3.396	4.436	4.255	4.425	3.43	3.023	3.498	3.749	3.628	3.07	3.429	1.72	2.011	1.341	1.705	1.704	1.115	0.49	0.76	1.208	3.789	2.299
	Cohen's d	1.242	1.119	1.105	1.142	1.042	0.849	1.109	1.064	1.106	0.857	0.756	0.875	0.937	0.907	0.767	0.857	0.430	0.503	0.335	0.426	0.426	0.279	0.122	0.190	0.302	0.947	0.575
1-gram	T(15)	-2.043	-0.594	-0.517	0.513	0.718	0.637	0.921	1.117	1.295	1.462	1.901	2.376	2.711	3.182	2.953	3.056	3.884	4.479	4.471	4.912	5.785	6.497	7.235	7.6	5.464	3.02	2.709
	Cohen's d	0.511	0.148	0.129	0.128	0.179	0.159	0.230	0.279	0.324	0.365	0.475	0.594	0.678	0.796	0.738	0.764	0.971	1.120	1.118	1.228	1.446	1.624	1.809	1.900	1.366	0.755	0.677
2-gram	T(15)	1.929	2.296	2.535	2.957	3.073	4.282	4.55	4.364	3.881	2.972	3.022	2.804	2.818	3.058	2.916	2.978	2.924	3.244	3.684	3.761	2.87	2.679	2.465	3.428	5.594	4.157	2.22
	Cohen's d	0.482	0.574	0.634	0.739	0.768	1.070	1.137	1.091	0.970	0.743	0.756	0.701	0.705	0.765	0.729	0.744	0.731	0.811	0.921	0.940	0.717	0.670	0.616	0.857	1.399	1.039	0.555
3-gram	T(15)	6.344	5.112	6.315	6.407	5.196	5.001	5.392	5.062	4.397	3.949	3.958	4.227	4.005	3.879	4.036	3.815	2.909	na	na	na	na	na	na	na	na	5.911	3.434
	Cohen's d	1.586	1.278	1.579	1.602	1.299	1.250	1.348	1.265	1.099	0.987	0.989	1.057	1.001	0.970	1.009	0.954	0.727	na	na	na	na	na	na	na	na	1.478	0.858

Note. No statistical tests were performed on "na" due to the ceiling effect.

P value of the Kolmogorov-Smirnov test

Rank		1	2	3	4	5	6	7	8	9	10	11	12	13	14	15	16	17	18	19	20	21	22	23	24	25	mean	proto
LD	same	0.066	0.633	0.633	0.633	0.912	0.912	0.348	0.633	0.162	0.912	0.348	0.633	0.633	0.912	0.162	0.348	0.633	0.348	0.348	0.633	0.999	0.633	0.912	0.633	0.633	0.348	0.999
	different	0.633	0.633	0.912	0.633	0.633	0.162	0.633	0.999	0.162	0.912	0.912	0.633	0.633	0.633	0.348	0.348	0.633	0.633	0.633	0.162	0.912	0.066	0.348	0.633	0.162	0.999	0.912
1-gram	same	0.912	0.633	0.912	0.912	0.348	0.912	0.162	0.912	0.633	0.633	0.999	0.912	0.999	0.912	0.999	0.348	0.348	0.633	0.912	0.348	0.633	0.912	0.348	0.348	0.633	0.633	0.912
	different	0.999	0.633	0.348	0.633	0.633	0.348	0.633	0.999	0.066	0.912	0.912	0.912	0.348	0.633	0.348	0.912	0.348	0.066	0.912	0.999	0.633	0.348	0.912	0.348	0.348	0.162	0.999
2-gram	same	0.999	0.066	0.348	0.912	0.348	0.633	0.348	0.912	0.912	0.912	0.633	0.912	0.912	0.162	0.912	0.912	0.633	0.912	0.912	0.633	0.348	0.912	0.162	0.066	0.348	0.162	0.912
	different	0.633	0.912	0.912	0.912	0.348	0.633	0.633	0.633	0.912	0.912	0.633	0.912	0.633	0.912	0.912	0.999	0.912	0.912	0.912	0.999	0.912	0.912	0.912	0.348	0.999	0.066	0.912
3-gram	same	0.912	0.633	0.912	0.348	0.912	0.912	0.912	0.633	0.912	0.348	0.633	0.633	0.633	0.162	0.633	0.912	0.912	0.912	0.348	0.633	0.633	0.162	0.633	0.162	0.007	0.912	0.912
	different	0.912	0.162	0.912	0.633	0.633	0.912	0.348	0.162	0.066	0.162	0.633	0.912	0.999	0.633	0.348	0.912	0.912	0.633	0.633	0.633	0.162	0.912	0.162	0.002	0.002	0.633	0.633

Note. Three values, at rank 24 and 25 of 3-gram where no t-tests were performed because of the ceiling effects, might not be normally distributed without multiple corrections.
However these non-normalities were eliminated after applying multiple corrections n = 27.
